# Mutual amplification of HNF4α and IL-1R1 composes an inflammatory circuit in *Helicobacter pylori* associated gastric carcinogenesis

**DOI:** 10.18632/oncotarget.7239

**Published:** 2016-02-08

**Authors:** Lin Ma, Jiping Zeng, Qing Guo, Xiuming Liang, Li Shen, Shuyan Li, Yundong Sun, Wenjuan Li, Shili Liu, Han Yu, Chunyan Chen, Jihui Jia

**Affiliations:** ^1^ Department of Microbiology/Key Laboratory for Experimental Teratology of Chinese Ministry of Education, School of Medicine, Shandong University, Jinan 250012, Shandong, PR China; ^2^ Department of Biochemistry, School of Medicine, Shandong University, Jinan 250012, Shandong, PR China; ^3^ Department of Nursing, Ningxia People's Hospital, Yinchuan 750021, Ningxia, PR China; ^4^ Department of Hematology, Qilu Hospital, Shandong University, Jinan 250012, Shandong, PR China

**Keywords:** HNF4α, IL-1R1, NF-κB, Hp, gastric carcinogenesis

## Abstract

*Helicobacter pylori* (Hp) is an environmental inducer of gastritis and gastric cancer (GC). The immune response to Hp and the associated changes in somatic gene expression are key determinants governing the transition from gastritis to GC. We show that hepatocyte nuclear factor 4α (HNF4α) is upregulated by Hp infection via NF-κB signaling and that its protein and mRNA levels are elevated in GC. HNF4α in turn stimulates expression of interleukin-1 receptor 1(IL-1R1), which amplifies the inflammatory response evoked by its ligand IL-1β. IL-1β/IL-1R1 activates NF-κB signaling, thereby increasing HNF4α expression and forming a feedback loop that sustains activation of the NF-κB pathway and drives the inflammation towards GC. Examination of clinical samples revealed that HNF4α and IL-1R1 levels increase with increasing severity of Hp-induced gastritis and reach their highest levels in GC. Co-expression of HNF4α and IL-1R1 was a crucial indicator of malignant transformation from gastritis to GC, and was associated with a poorer prognosis in GC patients. Disruption of the HNF4α/IL-1R1/IL-1β/NF-κB circuit during Hp infection maybe an effective means of preventing the associated GC.

## INTRODUCTION

Gastric cancer (GC) is a life-threatening malignancy and the fourth most common cancer worldwide [1]. *Helicobacter pylori* (Hp) infection, one of GC's main causes, leads to irreversible pathological changes in the gastric cavity. This bacteria activates multiple oncogenic signaling cascades, including MAPK, NF-κB, STAT3, and β-catenin pathways [2], which stimulate expression of a wide range of inflammatory genes, such as cytokines, chemokines, and adhesion molecules [3,4]. This process contributes to the aggravation of inflammatory responses, eventually inducing tissue damage. In contrast, patients with a sub-group of Hp-induced gastritis developed no final-stage uncontrolled malignancy, a result that suggests individual host response is a key contributor to gastric carcinogenesis [5, 6].

The nuclear receptor superfamily is a group of transcription factors that sense specific molecules, regulate gene expression, and play essential roles in inflammatory disorders [7]. Hepatocyte nuclear factor 4α(HNF4α), one member of the nuclear receptor superfamily, is expressed in liver, pancreas, stomach and colon [8]. Homodimeric HNF4α binds to specific DNA sequences, regulating a series of downstream genes involved in cell metabolism and differentiation [9–11]. HNF4α mutants account for maturity-onset diabetes of young people [12], and its deletion promotes the formation of hepatic carcinoma [13]. An HNF4α-microRNA circuit is perturbed in inflammation-related hepatic carcinogenesis [14]. On the other hand, replication of cancer-initiating virus HBV is dependent on expression of HNF4α [15]. HNF4α was identified as a susceptibility locus for ulcerative colitis and a tumorigenesis regulator in colon cancer [16, 17]. Further study of HNF4α is essential to unravel the close relationships between HNF4α and inflammatory neoplastic diseases.

While nuclear receptors control intracellular genomic transcription, cell surface receptors play important roles in communication between the cell and outside microenvironment. Interleukin-1 receptor 1(IL-1R1) signaling plays key roles in local inflammation and immune responses [18]. IL-1R activation through IL-1 binding causes nuclear localization of transcriptional activators, e.g. NF-κB, influencing various biological processes [19]. One of its ligands, Interleukin-1 beta [IL-1β], is a critical inflammation factor related to poor prognosis and patient survival in cancer [20]. In gastritis, deviant coupling of IL-1β and IL-1R plays a central role in the pathogenesis of Hp-induced mucosal inflammation [21].

In this study, we showed that HNF4α forms an inflammatory circuit with its direct target IL-1R1 in gastric carcinogenesis. Hp infection induces expression of HNF4α via the NF-κB pathway. HNF4α subsequently activates the expression of IL-1R1 and amplifies gastric cell inflammatory responses to IL-1β, which further stimulates the already over-active NF-κB pathway, completing a positive feedback loop that drives continuous inflammation. Nuclear receptor HNF4α and cell surface receptor IL-1R1 create an inflammation-perpetuating loop during Hp infection that holds potential in controlling gastric carcinogenesis.

## RESULTS

### HNF4α is up-regulated from gastritis to GC and its activity increases gastric cell proliferation

**Figure 1:**
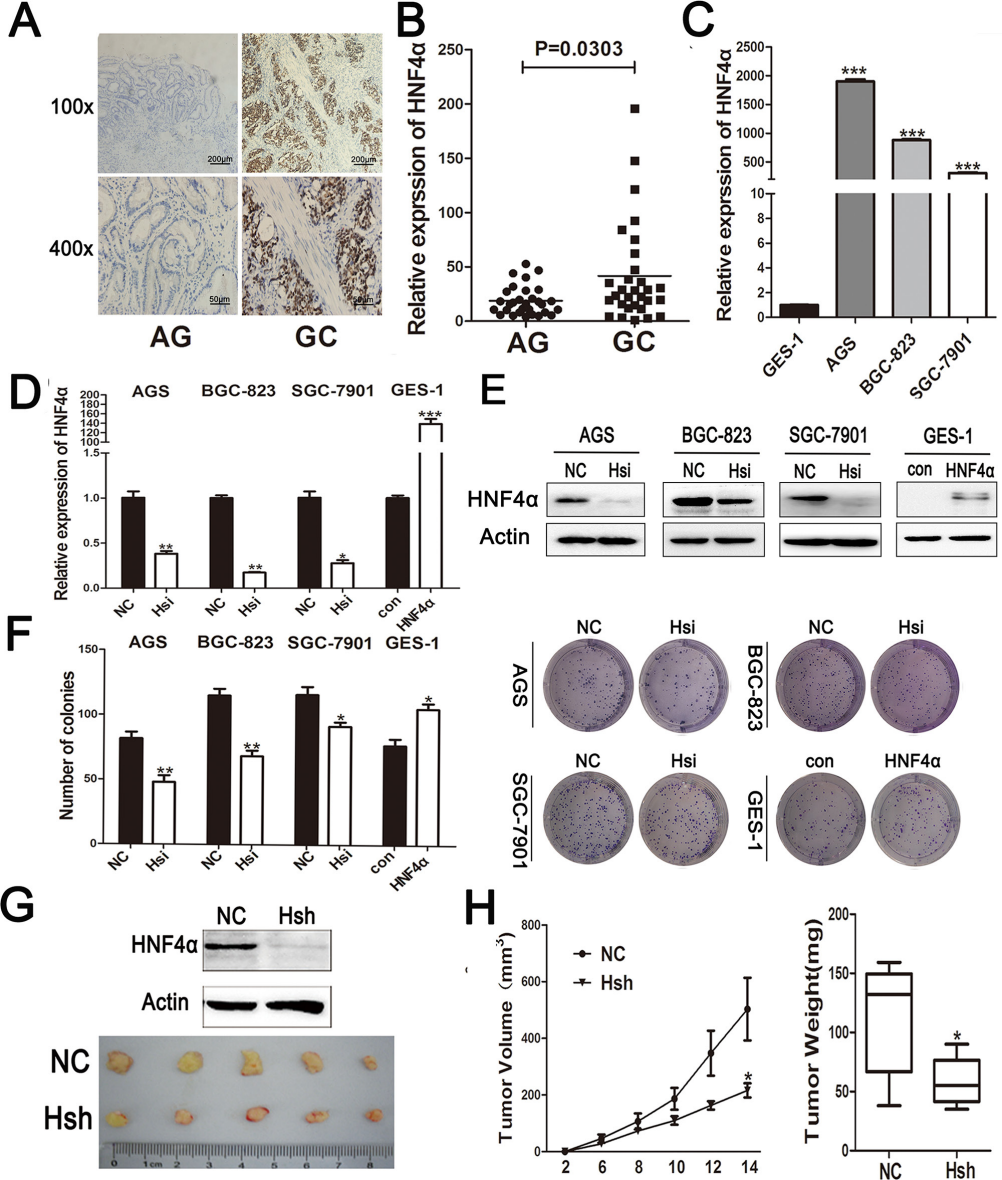
Expression ofHNF4α in clinical gastric tissues and its role in regulating gastric cell proliferation **A.** IHC staining of HNF4α in atrophic gastritis and gastric cancer samples. Representative images are shown here (magnification 100x,400x; Scale bars:200μm,50μm). **B.** The mRNA levels of HNF4α in 30 atrophic gastritis samples and 30 gastric cancers were measured by real-time PCR. The horizontal bars indicate the mean value of each sample group. Mann-Whitney *U*-test was used to calculate the P value. **C.** HNF4α mRNA expression levels in indicated gastric cancer cell lines and immortalized gastric cells GES-1. ***p<0.001 by Student's *t*-test. **D.** and **E.** HNF4α mRNA and protein levels in indicated cells transfected with HNF4α siRNA or HNF4α over-expression plasmid. *p<0.05, **p<0.01, ***p<0.001 by Student's *t*-test. **F.** Foci formation after cells transfected HNF4α siRNA or HNF4α over-expression plasmid. *p<0.05, **p<0.01 by Student's *t*-test. Representative images are shown here from three independent biological replicates. **G.** Western blot shows loss of HNF4α in BGC-823 cells transfected with lenti-HNF4α shRNA; Tumors formed in nude mice induced by BGC-823 cells transfected with lenti-negative control group(NC)and lenti-HNF4α shRNA group(Hsh). **H.**Tumor growth curve and tumor weight of NC group and Hsh group. *p<0.05 by Student's *t*-test.

To investigate nuclear receptor genes differentially expressed between gastritis and gastric cancer, we performed gene expression profiling on 3 atrophic gastritis samples and 3 gastric cancer samples. We combined our microarray data with the Human Protein Atlas database and selected 13 nuclear receptors and depicted their expression levels in a heat map diagram. HNF4α was increased in gastric cancer compared with atrophic gastritis samples (Supplementary Figure S1A). Immunohistochemisty (IHC) staining and real-time PCR analysis confirmed higher HNF4α expression in GC (Figure 1A and 1B). Additionally, HNF4α expression in gastric cancer cells, especially in AGS, BGC-823 and SGC-7901, was found to be higher than that in immortalized epithelial cell GES-1 (Figure 1C).

To determine the role of HNF4α in proliferation of gastric cell lines, we used siRNA to inhibit HNF4α expression in AGS, BGC-823, and SGC-7901 cells and over-expressed HNF4α with HNF4α-coding plasmid in GES-1 cells (Figure 1D and 1E). Colony formation assay results showed that knockdown of HNF4α in indicated GC cells decreased their colony formation, while over-expression of HNF4α promoted proliferation of GES-1 cells (Figure 1F). Consistent results were also independently observed with 5-ethynyl-2′-deoxyuridine (EdU) staining assay (Supplementary Figure S1B).

To confirm the *in vitro* finding that HNF4α knockdown inhibits GC cell proliferation, we transfected BGC-823 cells either with lenti-HNF4α shRNA or lenti-negative control and subcutaneously injected these cells into a mouse model. Tumor growth was significantly inhibited for cells with HNF4α knockdown (Hsh) compared to control group (NC) (Figure 1G and 1H). Our data showed that HNF4α expression was up-regulated in gastric carcinogenesis and its expression contributes to *in vitro* gastric cell proliferation and *in vivo* tumor growth.

### Hp infection promotes expression of HNF4α *in vitro* and *in vivo*

**Figure 2:**
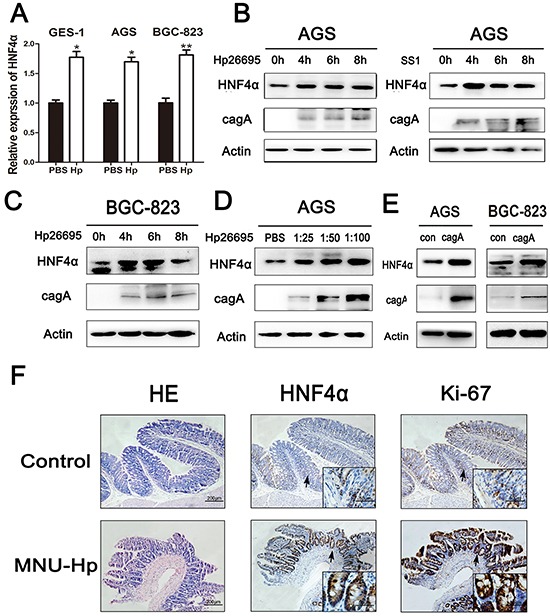
HNF4α expression level was up-regulated by Hpinfection *in vitro* and *in vivo* **A.** Real-time PCR shows mRNA levels of HNF4α in GES-1, AGS, and BGC-823 cell lines when cells were treated with PBS or Hp26695 for 4 hours respectively,*p<0.05,**p<0.01 by Student's *t*-test. **B.** and **C.** Western blot shows protein levels of HNF4α in AGS and BGC-823 cells incubated with Hp strains(26695 or SS1) for 4, 6, and 8 hours (multiplicity of infection (MOI) 1:100 (cell:Hp)) **D.** AGS cells were infected by Hp26695 with MOI at 1:25, 1:50, and 1:100 for 4 hours. Western blot indicates a gradual increasing trend in HNF4α. **E.** AGS and BGC-823 cells were transfected with cagA over-expression plasmid for 48 hours. Western blot shows increased protein level of HNF4α with cagA transfection. **F.** Hematoxylin and eosin (HE) staining and IHC assay of HNF4α and Ki-67 in mucosal epithelial tissue of control group and MNU-Hp infection group. Representative images are shown here (magnification: 100x, 400x; Scale bars:200μm, 50μm).

Hp infection is one of the major risk factors for gastric neoplasia [22]. ^13^C urea breath test was used to detect Hp infection in 30 patients with atrophic gastritis, and the level of HNF4α in Hp-positive patients was considerably higher than Hp-negative patients (Supplementary Figure S2A). To determine whether this bacterial infection could affect expression of HNF4α in gastric epithelial cell lines, GES-1, AGS, and BGC-823 cells were incubated with different Hp strains (26695 or SS1). Real-time PCR showed increased expression of HNF4α in indicated cells with addition of Hp26695 (Figure 2A). Hp26695 or SS1 also increased protein levels of HNF4α in AGS and BGC-823 cells at different time points (Figure 2B and 2C). HNF4α expression in AGS cells also showed a dose-dependent increase in response to Hp26695 (Figure 2D). CagA was one of the major virulence factors of Hp [23]. Exclusive over-expression of cagA was sufficient to promote production of HNF4α in AGS and BGC-823 cells (Figure 2E).

Hp inoculation has been shown to induce precancerous lesions in murine stomach [24]. To investigate the effect of Hp on expression of HNF4α *in vivo*, the above mouse model of Hp infection was performed. We decreased the dosage of MNU in drinking water of mice to better simulate the chronic impairment under natural conditions. HE staining showed severe atrophic mucosa with intestinal metaplasia in MNU-Hp group and normal gastric mucosa without inflammatory cell infiltration in control group. Interestingly, IHC confirmed that expression of HNF4α already showed a higher expression level in atrophic gastritis mucosa than normal mucosa (Figure 2F), along with a higher level of Ki-67 staining which nicely corroborated our previous finding that HNF4α contributed to gastric cell proliferation. The MNU group also developed severe atrophic mucosa, but the disease incidence and the expression of HNF4α were both to a less extent than MNU-Hp group (Supplementary Figure S2B and Supplementary Table S2). Thus, it indicated that Hp infection increases HNF4α expression before the tumor occurs and HNF4α may trigger the final transformation.

### Hp infection up-regulates HNF4α expression via NF-κB pathway activation and under transcriptional regulation of p65

**Figure 3:**
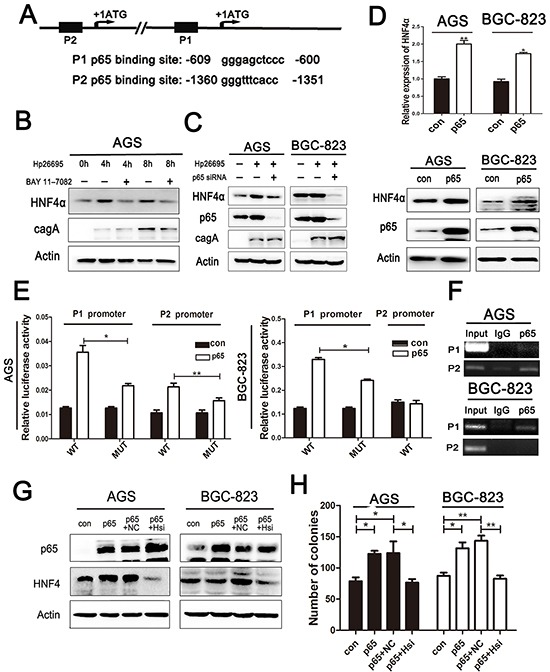
HNF4α induced by Hp is p65 dependent **A.** p65 binding sequence in HNF4αP1 and P2 promoter. **B.** Western blot shows HNF4α expression in AGS infected by Hp26695 with or without administration of BAY 11-7082(1.0 mM) **C.** Western blot shows HNF4α expression changes in AGSand BGC-823 cells infected by Hp26695 with or without transfection of p65 siRNA. **D.** RT-PCR and Western blot analysis of mRNA and protein levels of HNF4α expression in AGS and BGC-823 cells transfected with p65 over-expression plasmid. *p<0.05,**p<0.01 by Student's *t*-test. **E.** HNF4α P1 and P2 promoter activity increased with transfection of p65 over-expression plasmid in AGS cells, and only P1 promoter increased in BGC-823 cells. Mutation of p65 binding sequence reduced the induction effect of p65 on luciferase activity. **F.** ChIP assay confirmed the direct binding of p65 and HNF4αP1 and P2 promoter. Data are mean±SD of 3 biological replicates. **p<0.01,***p<0.001 by Student's t-test. **G.** Western blot indicates protein level of p65 and HNF4α in AGS and BGC-823 cells transfected with p65 plasmid and HNF4α siRNA solely or jointly. **H.** Attenuation of pro-foci formation effects of p65 by knockdown of HNF4α expression in AGS and BGC-823 cells,*p<0.05,**p<0.01 by Student's *t*-test.

To further unravel the mechanism by which Hp infection induces HNF4α expression, we analyzed the nucleotide sequence of HNF4α promoter for putative transcription factor binding sites. HNF4α isoforms are generated by alternative regulating promoters, P1 and P2 [25], and we found that both P1 and P2 promoters contain a conserved p65 binding motif as has been reported (Figure 3A) [26, 27]. We used BAY 11-7082 to inhibit nuclear translocation of p65 or p65 siRNA to knockdown the expression of p65 before addition of Hp, and found that both BAY 11-7082 and p65 siRNA reversed the induction of HNF4α expression by Hp in AGS and BGC-823 cells (Figure 3B and 3C). To further confirm the result, we transfected cells with p65 plasmid. Over-expression of p65 markedly increased HNF4α transcription and expression (Figure 3D).

Interestingly, in AGS cells, both P1 and P2 functions were detected with P2 promoter in dominance, while in BGC823 cells, only P1 function was detected (Supplementary Figure S3). To specifically prove that p65 transcriptionally activates P1 and P2 promoters of HNF4α, we constructed luciferase reporter plasmids containing P1 or P2 promoter (P1-WT and P2-WT), respectively. Consistent with aforementioned results, over-expression of p65 increased luciferase signals in both P1-WT and P2-WT transfected AGS cells, but only increased P1-WT but not P2-WT transfected BGC-823 cells. Then constructs with mutated p65 binding sites, P1-MUT and P2-MUT, were tested and both showed diminished luciferase signals compared to WT promoters, though increased luciferase activity after p65 over-expression was still observed (Figure 3E). This indicates that some indirect regulation of HNF4α by p65 may exist. To directly validate association of p65 with HNF4α promoters, we conducted ChIP analysis in AGS and BGC-823 cells. ChIP results showed that in AGS cells, p65 binds more avidly to P2 than to P1 promoter, but mainly binds to P1 promoter in BGC-823 cells (Figure 3F), which corroborated our previous findings.

As shown above, HNF4α had a significant impact on cell proliferation in gastric cancer cell lines. P65 was also implicated in tumor cell proliferation [28]. HNF4α knockdown abrogated the promoting effects of p65 on cell proliferation in both AGS and BGC-823 cells, which indicated that cell proliferation induced by p65 was at least partly depended on HNF4α expression (Figure 3G and 3H).

### IL-1R1 is up-regulated from gastritis to GC and is the direct target of HNF4α

**Figure 4:**
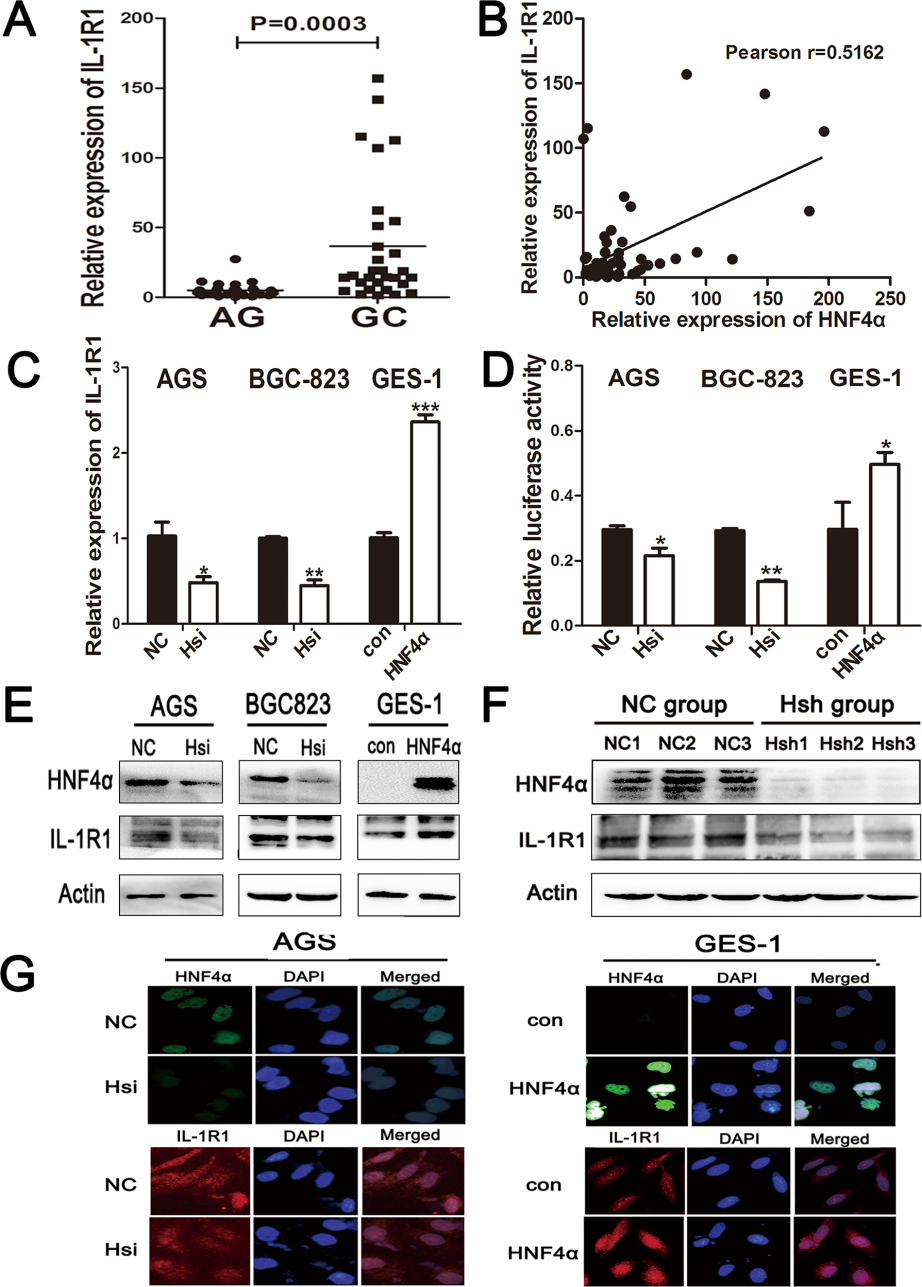
IL-1R1 is up-regulated in gastric cancer tissues and it is the direct target of HNF4α **A.** The mRNA levels of IL-1R1 in 30 atrophic gastritis samples and 30 gastric cancers were measured by real-time PCR. The horizontal bar indicates the mean value of each sample group. Mann-Whitney *U*-test is used to calculate the P value. **B.** Positive correlation between HNF4α and IL-1R1 expression in the above tissues. Each data point represents an individual gastric tissue sample, and a correlation coefficient r is shown. **C.** RT-PCR indicates IL-R1 mRNA levels in indicated cells transfected with HNF4α siRNA and HNF4α over-expression plasmid. **D.** Luciferase reporter assay confirmed the direct interaction between HNF4α and IL-1R1 promoter. Data are mean±SD of 3 biological replicates. *p<0.05,**p<0.01,***p<0.001 by Student's *t*-test. **E.** and **G.** IL-R1 protein level changes in indicated cells transfected with HNF4α siRNA and HNF4α over-expression plasmid using western blot and immunofluorescence. **F.** Western blot shows HNF4αand IL-1R1 protein expression in tissues from nude mice in NC group and Hsh group.

Hp infection can bring microenvironmental change to gastric mucosa, including infiltration of inflammatory cells and accumulation of cytokines. Since cell surface receptors control communication with the outside environment and can modify important cellular functions, we compared expression of 21 cell surface receptors between atrophic gastritis and gastric cancer, and identified IL-1R1 as most significantly increased in GC (Supplementary Figure S4A). Real-time PCR showed higher expression of IL-1R1 in GC than atrophic gastritis, and IL-1R1's expression level was also connected with Hp infection (Figure 4A and Supplementary Figure S4B). We found a positive correlation between the mRNA levels of HNF4α and IL-1R1 in the same gastric samples (Figure 4B). IL-1R1 mRNA and protein levels were significantly decreased when HNF4α was knocked down in AGS and BGC-823 cells, and over-expression of HNF4α led to elevated IL-1R1 levels in GES-1 cells (Figure 4C, 4E and 4G). We also created a luciferase reporter plasmid with inserts of IL-1R1 promoter region containing HNF4α binding sequence. Knockdown of HNF4α inhibited luciferase signals with a construct containing the IL-1R1 promoter and vice versa (Figure 4D). To further confirm the regulation of IL-1R1 by HNF4α *in vivo*, we performed western blot using tumor samples from HNF4α shRNA and negative control groups as previously described. Both HNF4α and IL-1R1 levels were decreased in Hsh group compared with NC group (Figure 4F). The above results imply that HNF4α regulates IL-1R1 both *in vitro* and *in vivo*.

### HNF4α and IL-1R1 is essential for sustained activation of NF-κB pathway in IL-1β-associated inflammation

**Figure 5:**
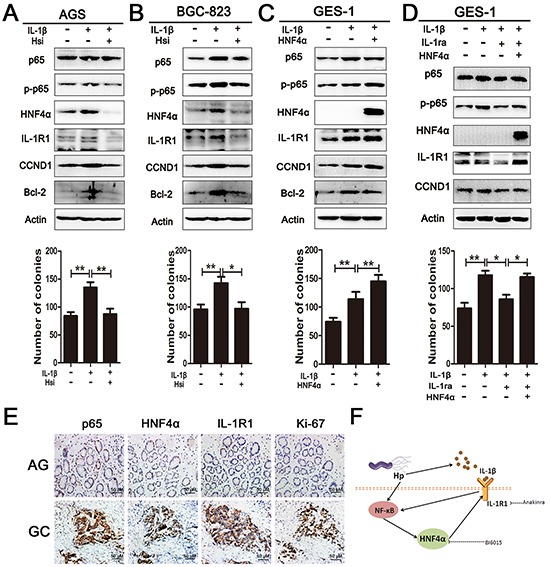
HNF4α and IL-1R1 is essential for IL-1β-induced expression and phosphorylation of p65 **A.** and **B.** Western blot shows pre-treatment with HNF4α siRNA in AGS and BGC-823 cells decreased p65 expression and phosphorylation induced by IL-1β(10ng/ml). Foci formation assay showed knock down of HNF4α attenuates the pro-foci formation effect of IL-1β in AGS and BGC-823 cells. *p<0.05,**p<0.01 by Student's *t*-test. **C.** Western blot and foci formation assay shows HNF4α over-expression markedly increased p65 phosphorylation and foci formation induced by IL-1β in GES-1 cell lines. **D.** Western blot sand foci formation assay shows HNF4α over-expression in GES-1 cells retrieved IL-1β transduction when IL-1R1 was blockaded by IL-1RA (500ng/ml). **E.** p65, HNF4α, IL-1R1, and ki67 expression in serial sections from atrophic gastritis and gastric cancer. Representative images are shown here (magnification 400x;Scale bars:50μm). **F.** Schematic representation of the proposed HNF4α and IL-1R1 feedback circuit in Hp associated gastric oncogenesis.

IL-1β, a ligand of IL-1R1, is well known as a pro-inflammatory cytokine and its over-expression is an essential risk factor in gastric cancer [29]. It is also a potent inducer of NF-κB activity [30]. Our data indicated that Hp infection elicits IL-1β release by THP-1 monocytes (Supplementary Figure S5A). Addition of exogenous IL-1β(10ng/ml) at various time points increased expression of p65, HNF4α, and IL-1R1 in cultured AGS and BGC-823 cells (Supplementary Figure S5B). Administration of BAY 11-7082, a translocation inhibitor of p65, diminished the effect of IL-1β (Supplementary Figure S5C). It was reported that IL-1β induced phosphorylation of p65 at serine 536 and activated NF-κB-dependent transcription [31]. Since HNF4α directly regulates expression of IL-1R1, we wondered whether alteration of HNF4α changed the cellular response to IL-1β. Knockdown of HNF4α in AGS and BGC-823 cells decreased p65 expression and p65 s536 phosphorylation as expected. The same trends were observed in p65 direct downstream target genes, Bcl-2 and CCND1. Inhibition of HNF4α also reduced the pro-proliferation effect of IL-1β (Figure 5A and 5B). As shown in Figure 5C, in response to IL-1β stimulation, over-expression of HNF4α increased p65 expression, p65 s536 phosphorylation, and cell proliferation ability compared to sham group. Interleukin-1 receptor antagonist (IL-1RA) can compete with IL-1β at the receptor level and block IL-1R1 signaling [32]. Treatment with IL-1RA (500ng/ml) attenuated IL-1β induced increases in p65 expression and p65 s536 phosphorylation, while over-expression of HNF4α led to increased expression of IL-1R1 and restored capacity of IL-1β signal transduction (Figure 5D). IHC staining in serial sections revealed co-expression of p65, HNF4α, IL-1R1, and ki67 in progression of gastric carcinogenesis (Figure 5E). Thus, our data support that HNF4α and its target IL-1R1 play an essential role in amplifying IL-1β-associated inflammation.

### Expression of HNF4α and IL-1R1 in gastric carcinogenesis and their co-expression indicates poor prognosis for GC

**Figure 6:**
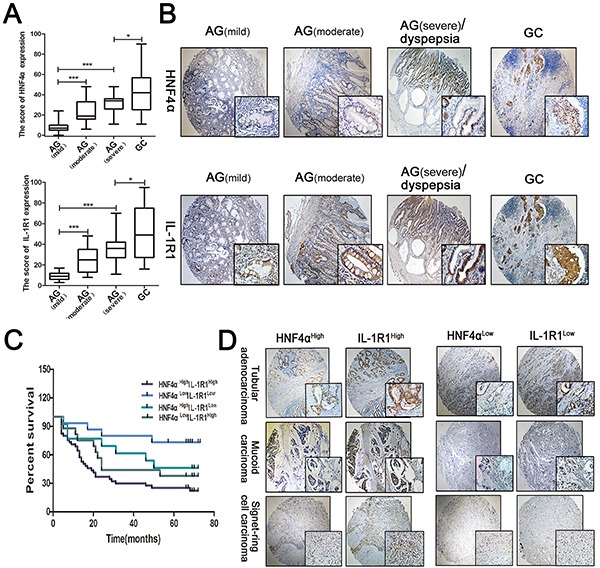
Expression of HNF4α and IL-1R1 in clinical gastric samples **A.** Box plots indicate the IHC score of HNF4α and IL-1R1 in different stages of gastritis and gastric cancer. The line in each box represents the median. *p<0.05, **p<0.01,***p<0.001 by Mann-Whitney *U*-test. **B.** IHC staining showed HNF4α and IL-1R1 were both up-regulated in the progression of gastric cancer. Representative images are shown here(magnification 40x, 400x). **C.** Kaplan-Meier survival curves for survival (months) of 90 gastric cancer patients stratified by HNF4α and IL-1R1 expression, including HNF4α^High^IL-1R1^High^(n=45), HNF4α^Low^IL-1R1^Low^(n=15), HNF4α^High^IL-1R1^Low^(n=13), and HNF4α^Low^Il-1R1^High^(n=17). The HNF4α^High^IL-1R1^High^ group had poorer outcomes than the HNF4α^Low^IL-1R1^Low^ group (p=0.0011) **D.** Representative cases of different types of gastric cancer in HNF4α^High^IL-1R1^High^ group and HNF4α^Low^IL-1R1^Low^ group. Magnification 40x, 400x.

We collected 75 atrophic gastritis and 160 gastric cancer samples from patients and divided the 75 atrophic gastritis into mild (n=31), moderate (n=25), and severe (n=19) categories depending on their clinical diagnoses. IHC staining in serial sections revealed co-expression of HNF4α and IL-1R1 which gradually increases with progression of disease. HNF4α and IL-1R1 showed an increasing trend along with the severity of atrophic gastritis already, which was in accordance with the results in mice, and were increased in gastric cancer samples (Figure 6A and 6B).

We further examined HNF4α and IL-1R1 expression in 90 gastric cancer samples and corresponding normal tissues. Survival curves were plotted to compare patient outcomes in different expression levels of HNF4α and IL-1R1 and we found a reduction of survival time in patients with high co-expression levels of HNF4α and IL-1R1 in different types of gastric cancer (Figure 6C and 6D). Overall, these data reveal that co-expression of HNF4α and IL-1R1 serves as a biomarker in transformation from gastritis to GC and their co-expression indicates worse prognosis of GC.

## DISCUSSION

Our results reveal NF-κB, HNF4α and IL-1R1 are all part of the self-perpetuating circuit connecting gastritis to gastric cancer. First, NF-κB activation leads to HNF4α over-expression after Hp infection. NF-κB p65 subunit directly binds to the HNF4α, P1 and P2 promoters and mediates the HNF4α expression increase in Hp infection. Second, HNF4α regulates the expression of IL-1R1. This study proves HNF4α is a positive regulator of IL-1R1 and amplifies the epithelial cellular response to IL-1β, representative of local inflammatory cytokines. Lastly, via up-regulated IL-1R1 receptor, Hp infection-induced expression of IL-1β further activates the NF-κB signaling pathway and completes the self-reinforcing feedback loop. Persistence of the circuit results in prolonged activation of HNF4α, uncontrolled cell proliferation, and, eventually, cancer progression.

Other research groups have shown HNF4α plays a critical role in inflammation-associated cancers and exhibits certain organ specificity. Of course, controversy about the powers of HNF4α in regard to cancer challenges the belief that HNF4α promotes cancer-induction. Recent findings suggest an inhibitory effect by HNF4α on hepatic carcinoma progression, which was attributed to inhibition of the Wnt/β-catenin signaling pathway [33]. NF-κB activation represses HNF4α expression via induction of miR-21 in hepatic tumors [34].

On the other hand, HNF4α was reported to facilitate the initiation of intestinal cancer in the Apc^Min^ mouse model [35]. In line with our results, we found HNF4α played an oncogenic role in gastric carcinogenesis and was positively regulated by the NF-κB pathway. In fact, the different immune responses in liver verses the gastrointestinal cavities may account for the differential roles of HNF4α and NF-κB's regulation of carcinogenesis in those organs. HNF4α can be a therapeutic target in early stage of gastric cancer [36]. Our data indicate that HNF4α functions as an amplifier in infection-induced inflammation and as an oncogene even before the early GC stages, during the transformation from gastritis to GC. HNF4α expression is increased in atrophic gastritis and, upon malignant transformation, reaches an even higher level. As our research deepens the understanding of HNF4α's involvement in the progression through various stages of gastric cancer, including the changes in expression pattern and the variation of isoforms, HNF4α emerges as a possible game-changing molecule that requires further study.

It is well known that IL-1and IL-1R1 signaling plays an essential role in inflammatory disease. As a cell surface receptor, IL-1R is responsible for cell communication with the outside microenvironment. Activation of IL-1 in both paracrine and autocrine fashions was observed in stomach samples from Hp-infected patients. During infection, binding of cytokine IL-1 to IL-1R1 enables transmigration and infiltration of inflammatory cells and over-activation leads to tissue damage [37]. IL-1R^−/−^ mice were protected from *Helicobacter felis*-induced gastritis and pre-neoplastic pathology was absent in these mice [38]. Besides IL-1and IL-1R1, many other cytokines and associated receptors, for example IL-6 and TNFα, are involved in the progression of gastric cancer and can activate NF-κB signaling. We have examined many cell surface receptors after HNF4α knockdown in gastric cancer cell lines. Because of low expression of IL-6R and IL-8R in gastric cells, there was no obvious gene expression change of IL-6R and IL-8R. The decrease in levels of TNFR1 and IL-18R was much less pronounced than IL-1R1. In our study, we connected HNF4α, a nuclear gene expression regulator, with IL-1R1. These findings delineate the cell communication changes resulting from Hp infection.

NF-κB activation, which leads to aberrant cell proliferation, resistance to apoptosis, angiogenesis, and tumor metastasis, is central in tumor progression [39–41]. Three bacterial products were thought major activators of NF-κB in Hp infection: LPS, peptidoglycan, and cagA [42]. Despite its essential role in inflammation and immunity, activation of NF-κB is a double-edged sword. On one hand, NF-κB is essential for optimal activation of innate and adaptive immune responses against pathogens; on the other, sustained, constitutive NF-κB activation results in irreversible pathologic changes [43]. Therefore, activated NF-κB signals need to be properly switched off in case of prolonged and detrimental inflammatory responses. Our data insinuates that, due to the dual receptor response to Hp infection composed by nuclear receptor HNF4α and its target IL-1R1, a combined therapeutic strategy might be more efficient in controlling NF-κB activation for Hp associated gastric diseases. Simultaneous inhibition of HNF4α and IL-1R1 would better interrupt the pathogenic feedback cycle and retain immune homeostasis in the local microenvironment [Figure 5F]. As clinical treatments have already using IL-1Ra recombinant agent Anakinra, and BIM5078 and BI6015 are HNF4α antagonists, it is highly possible that combination therapy using antagonists with Anakinra show simultaneous inhibition of HNF4α and IL-1R1 [44, 45].

Taken together, our findings proved a self-perpetuating circuit of HNF4α and its direct target IL-1R1 after Hp infection via sustained activation of NF-κB pathway. This contributes to a deeper understanding of the linkage between Hp infection and eventual inflammation-related progression into gastric cancer. It also provides an innovative perspective concerning combined therapy on better prevention and treatment of Hp infection induced gastric diseases.

## MATERIALS AND METHODS

### Cell culture and transfection

The immortal gastric epithelium cells GES-1 and gastric cancer cells AGS, BGC-823, and SGC-7901 were cultured in RPMI-1640 containing 10% fetal bovine serum. Cell lines expressing HNF4α shRNA were developed by infecting BGC-823 cells with the specific virus and selected with 4μg/ml Puromycin. All cells were maintained in a humidified 5% CO_2_ incubator. Cell transfection was performed using Lipofectamine 2000 (Invitrogen) according to the manufacturer's protocol.

### Microarray analysis

For the analysis of differential gene expression in atrophic gastritis and gastric cancer, NimbleGen One-Color DNA Labeling Kit was used for sample cDNA labeling. Hybridization was performed using the NimbleGen Hybridization System. After washing, slides were scanned with an Axon GenePix 4000B scanner. Data was extracted and normalized using NimbleScan v2.5 software. Further data analysis was performed using Agilent GeneSpring GX 11.0 software.

### Regents and transfection

Constructs: HNF4α siRNA (Sigma-aldrich), HNF4α shRNA constructed in pLV3 vector (GenePharma), Bay 11-7082 (Sigma-aldrichInc), MNU(Sigma-aldrich), Recombinant Human IL-1β(Peprotech), Recombinant Human IL-1RA(Peprotech);

Antibodies: HNF4α(CST), p65(Abcam), IL-1R1(Santa Cruz), p-p65(Cell Signaling), cagA(Abcam), ki67(Abcam)CCND1(Cell Signaling), Bcl-2(Santa Cruz), b-actin(Sigma aldrich).

### Patient samples

RNA samples: RNAs from 30 AG and 30 GC were collected immediately followingoscopic biopsy or surgery and stored in RNAlater(QIAGEN) at −80°C until RNA extraction. FFPE samples: 75 samples of AG and 160 samples of GC were collected immediately after endoscopic biopsy or surgery and stored in formalin. The diagnosis for all patients was confirmed by histological examination. General characteristics of patients are shown in Table 1.

**Table 1:** Clinical characteristics of gastric samples from AG and GC patients

Clinical data	RNA samples			FFPE samples		
	AG(30)			AG(75)		
	HNF4α^Low^	HNF4α^High^	p	HNF4α^Low^	HNF4α^HIgh^	p
**Age**						
<median age	7	5	0.7116	20	6	0.5938
>median age	12	6		34	15	
**Gender**						
Male	13	8	1.0000	40	15	1.0000
Female	6	3		14	6	
**Pathological grade**						
Mild / Moderate	18	6	0.0156	49	7	<0.0001
Severe	1	5		5	14	
**IL-1R1 expression**						
IL-1R1^Low^	14	3	0.0224	45	5	<0.0001
IL-1R1^High^	5	8		9	16	
	**GC(30)**			**GC(160)**		
	**HNF4α^Low^**	**HNF4α^High^**	**p**	**HNF4α^Low^**	**HNF4α^HIgh^**	**p**
**Age**						
<median age	5	7	0.4611	15	26	1.0000
>median age	5	13		43	76	
**Gender**						
Male	8	15	1.0000	38	53	0.1008
Female	2	5		20	49	
**TNM stage**						
I/II	7	5	0.0450	35	43	0.0328
III/IV	3	15		23	59	
**IL-1R1 expression**						
IL-1R1^Low^	5	2	0.0256	28	24	0.0016
IL-1R1^High^	5	18		30	78	

### Hp culture and mouse model of *H. pylori* infection

Hp strains 26695 and SS1 were grown in Brucella broth with 5% fetal bovine serum under microaerobic conditions (5% O2, 10% CO2, and 85% N2) at 37°C, harvested by centrifugation, and added to gastric cell lines at different bacteria-to-cell ratios and times. 48 male C57BL/6 mice 6∼8 weeks old were divided into 3 groups. Groups 1 and 2 were given MNU(30ppm)in their drinking water for 10 weeks. Then they were given distilled water for 2 weeks after administration of MNU. Then, Group 1 was inoculated with SS1 strains (1×109 colony-forming U/ml) every other day for a total of 3 times. 12 mice in Group 3 were given distilled water without MNU or *H. pylori* as controls. All mice were sacrificed at 50 weeks for further study.

### Tumor xenograft model

10 male thymus-null BALB/c nude mice were purchased from QING ZI LAN Animal Company (Nanjing, China) and divided into 2 groups. 4×10^5^BGC-823 cells transfected with lenti-HNF4α shRNA or lenti-negative control were injected subcutaneously in the nude mice. Tumor growth was monitored every 2 days for a total period of 14 days.

### Colony formation assay

Cells (500/well) were seeded in 6-well plates after they were subjected to the corresponding treatment. After 10∼15 days incubation, the cells were fixed with methanol and stained with Giemsa. The experiment was repeated three times and colonies with more than 50 cells were counted for the following analysis.

### EdU staining

DNA synthesis was determined using the Cell-Light™ EdU Apollo®488 *In Vitro* Imaging Kit according to the manufacturer's instructions (Guangzhou RiboBio).

### Reporter plasmid construction and luciferase assay

Human HNF4α P1 and P2 promoters and IL-1R1 promoter fragments were cloned from human genome DNA by PCR. The primer sequences, each of which contains KpnI and HindIII cutting sites respectively, can be seen in Supplementary Table S1. Fragments were inserted into the pGL3 Basic luciferase reporter vector (Promega). Luciferase reporter activity was measured by using the Luciferase Assay System (Promega) according to the manufacturer's instructions and relative luciferase activity was calculated by normalizing the firefly luminescence with to the renilla luminescence.

### Chromatin immunoprecipitation (ChIP) assay

The ChIP assay was conducted using Chromatin Immunoprecipitation (ChIP) Assay Kit (Mllipore). Briefly, the chromatin fragments derived from AGS and BGC-823 cells were immunoprecipitated with 5 ug of antibody against p65. The consequent precipitated DNA samples were detected with PCR; primers used in this experiment can be seen in Supplementary Table S1.

### RNA extraction, RT-PCR, and real-time PCR

Total RNA from cells under different treatments were extracted with Trizol Reagent according to the protocol and then reverse-transcribed with RevertAid First Strand DNA Synthesis kit (Fermentas) to form cDNA. The cDNAs were subjected to SYBR Green based real-time PCR analysis. Primers used in this experiment can be seen in Supplementary Table S1. HNF4α and IL-1R1 mRNA levels in patient samples were divided into 2 groups “high expression” and “low expression”, based on median expression in all samples.

### Western blotting

Cells were lysed in protein lysis buffer with protease and phosphatase inhibitors. Lysates were separated on SDS-PAGE gels and transferred to PVDF membranes, which were blocked with 5% nonfat dry milk and probed with specific primary antibodies followed by corresponding horseradish peroxidase-labeled secondary antibodies. Immunoblots were detected with Millipore ECL regents.

### Immunofluorescence

Cells were seeded on a sterile coverslip in a 6-well microtiter plate. After transfection, cells were fixed with 4% paraformaldehyde and incubated with corresponding antibodies overnight at 4°C. Anti-rabbit IgG (H+L) and F(ab')2 Fragment(CST) were used as secondary antibody the next day and nuclei were stained with DAPI(4-6-Diamidino-2-phenylindole dihydrochloride). We used an OLYMPUS fluorescence microscope to measure fluorescent signals.

### Immunohistochemistry

FFPE sections from patients were subjected to deparaffination and dehydration. After epitope retrieval and H_2_O_2_ treatment, sections were blocked in 5% normal goat serum for 30 min and incubated with specific antibodies overnight at 4°C. Slides were incubated with secondary antibody and detected using DAB staining kit (Vector Laboratories) the next day.

An IHC score for each case was calculated by multiplying staining intensity with the percentage of positive cells, resulting in an overall score between 0 and 100. Duplicate readings gave similar results. Samples with IHC score≤30 were considered low expression and those with IHC score>30 were considered high expression.

### Statistical analysis

All data from experiments with biological replicates are expressed as mean (±SD). Analysis was assessed by Student's t-tests, Mann-Whitney U-test, Pearson correlation efficiency analysis, Kaplan-Meier method, and the log-rank test. P values < 0.05 were considered statistically significant.

## SUPPLEMENTARY TABLES AND FIGURES



## References

[1] Jemal A, Bray F, Center MM, Ferlay J, Ward E, Forman D (2011). Global cancer statistics. CA Cancer J Clin.

[2] Ding SZ1, Goldberg JB, Hatakeyama M (2010). Helicobacter pyloriinfection, oncogenic pathwaysand epigenetic mechanisms in gastric carcinogenesis. Future Oncol.

[3] Houghton JeanMarie, Wang Timothy C. (2005). Helicobacter pylori and Gastric Cancer: A new paradigm for inflammation-Associated epithelial cancers. Gastroenterology.

[4] Fox JG1, Wang TC (2007). Inflammation, atrophy, and gastric cancer. J Clin Invest.

[5] Fuccio L, Zagari RM, Eusebi LH, Laterza L, Cennamo V, Ceroni L, Grilli D, Bazzoli F (2009). Meta-analysis: can Helicobacter pylori eradication treatment reduce the risk for gastric cancer?. Ann Intern Med.

[6] McColl KE (2010). Clinical practice. Helicobacter pylori infection. N Engl J Med.

[7] Glass CK, Saijo K (2010). Nuclear receptor transrepression pathways that regulate inflammation in macrophages and T cells. Nat Rev Immunol.

[8] Chen WS, Manova K, Weinstein DC, Duncan SA, Plump AS (1994). Disruption of the HNF-4 gene, expressed in visceral endoderm, leads to cell death in embryonic ectoderm and impaired gastrulation of mouse embryos. Genes De.

[9] Bogan AA, Dallas-Yang Q, Ruse MD, Maeda Y, Jiang G, Nepomuceno L (2000). Analysis of protein dimerization and ligand binding of orphan receptor HNF4alpha. J Mol Biol.

[10] Wang H, Maechler P, Antinozzi PA, Hagenfeldt KA, Wollheim CB (2000). Hepatocyte nuclear factor 4alpha regulates the expression of pancreatic beta-cell genes implicated in glucose metabolism and nutrient-induced insulin secretion. J Biol Chem.

[11] Bolotin E, Liao H, Ta TC, Yang C, Hwang-Verslues W, Evans JR, Jiang T, Sladek FM (2010). Integrated approach for the identification of human hepatocyte nuclear factor 4alpha target genes using protein binding microarrays. Hepatology.

[12] Stoffel M1, Duncan SA (1997). The maturity-onset diabetes of the young (MODY1) transcription factor HNF4alpha regulates expression of genes required for glucose transport and metabolism. ProcNatlAcadSci U S A.

[13] Walesky C, Edwards G, Borude P, Gunewardena S, O'Neil M, Yoo B, Apte U (2013). Hepatocyte nuclear factor 4 alpha deletion promotes diethylnitrosamine-induced hepatocellular carcinoma in rodents. Hepatology.

[14] Hatziapostolou M, Polytarchou C, Aggelidou E, Drakaki A, Poultsides GA, Jaeger SA, Ogata H, Karin M, Struhl K, Hadzopoulou-Cladaras M, Iliopoulos D (2011). An HNF4α-miRNA inflammatory feedback circuit regulate hepatocellular oncogenesis. Cell.

[15] Li L, Oropeza CE, Sainz B, Uprichard SL, Gonzalez FJ, McLachlan A (2009). Developmental regulation of hepatitis B virus biosynthesis by hepatocyte nuclear factor 4alpha. PLoS One.

[16] Barrett JC, Lee JC, Lees CW, Prescott NJ, Anderson CA, Phillips A, Wesley E, Parnell K, Zhang H, Drummond H, Nimmo ER, Massey D, UK IBD Genetics Consortium (2009). Genomewide association study of ulcerative colitis identifies three new susceptibility loci, including the HNF4A region. Nat. Genet.

[17] Darsigny M, Babeu JP, Seidman EG, Gendron FP, Levy E, Carrier J, Perreault N, Boudreau F (2010). Hepatocyte nuclear factor-4alpha promotes gut neoplasia in mice and protects against the production of reactive oxygen species. Cancer Res.

[18] O'Neill LA (2008). The interleukin-1 receptor/Toll-like receptor superfamily: 10 years of progress. Immunol Rev.

[19] Vallabhapurapu S, Karin M (2009). Regulation and function of NF-kappaB transcription factors in the immune system. Annu Rev Immunol.

[20] Deans DA, Wigmore SJ, Gilmour H, Paterson-Brown S, Ross JA, Fearon KC (2006). Elevated tumour interleukin-1beta is associated with systemic inflammation: A marker of reduced survival in gastro-oesophageal cancer. Br J Cancer.

[21] Yamaoka Y, Kita M, Kodama T, Sawai N, Kashima K, Imanishi J (1997). Induction of various cytokines and development of severe mucosal inflammation by cagA gene positive Helicobacter pylori strains. Gut.

[22] Peek RM, Crabtree JE (2006). Helicobacter infection and gastric neoplasia. J. Pathol.

[23] Hatakeyama M (2004). Oncogenic mechanisms of the Helicobacter pylori CagA protein. Nat Rev Cancer.

[24] Nam KT, Hahm KB, Oh SY, Ishida K, Kawasaki S, Katsuyama T, Shimizu N, Tatematsu M (2004). The selective cyclooxygenase-2 inhibitor nimesulide prevents Helicobacter pylori-associated gastric cancer development in a mouse model. Clin Cancer Res.

[25] Nakhei H, Lingott A, Lemm I, Ryffel GU (1998). An alternative splice variant of the tissue specific transcription factor HNF4alpha predominates in undifferentiated murine cell types. Nucleic Acids Res.

[26] Ai L, Skehan RR, Saydi J, Lin T, Brown KD (2012). Ataxia-telangiectasia, mutated (ATM)/Nuclear Factor κ light chain enhancer of activated B cells (NFκB) signaling controls basal and DNA damage-induced transglutaminase 2 expression. J Biol Chem.

[27] Ai L, Skehan RR, Saydi J, Lin T, Brown KD (2011). Genomic analysis reveals a novel nuclear factor-κB (NF-κB)-binding site in Alu-repetitive elements. J Biol Chem.

[28] Whitfield ML, George LK, Grant GD, Perou CM (2006). Common markers of proliferation. Nat Rev Cancer.

[29] Dinarello CA (2009). Interleukin-1beta and the autoinflammatory diseases. N Engl J Med.

[30] Vallabhapurapu S, Karin M (2009). Regulation and function of NF-kappaB transcription factors in the immune system. Annu Rev Immunol.

[31] Buss H, Dörrie A, Schmitz ML, Hoffmann E, Resch K, Kracht M (2004). Constitutive and interleukin-1-inducible phosphorylation of p65 NF-{kappa}B at serine 536 ismediated by multiple protein kinases including I{kappa}B kinase (IKK)-{alpha}, IKK{beta}, IKK{epsilon}, TRAF family member-associated (TANK)-binding kinase 1 (TBK1), and anunknown kinase and couples p65 to TATA-binding protein-associated factor II31-mediatedinterleukin-8 transcription. J Biol Chem.

[32] Arend WP, Malyak M, Guthridge CJ, Gabay C (1998). Interleukin-1 receptor antagonist: role in biology. Annu Rev Immunol.

[33] Ning BF, Ding J, Yin C, Zhong W, Wu K, Zeng X, Yang W, Chen YX, Zhang JP, Zhang X, Wang HY, Xie WF (2010). Hepatocyte nuclear factor 4 alpha suppresses the development of hepatocellular carcinoma. Cancer Res.

[34] Ning BF, Ding J, Liu J, Yin C, Xu WP, Cong WM, Zhang Q, Chen F, Han T, Deng X, Wang PQ, Jiang CF, Zhang JP (2014). Hepatocyte nuclear factor 4α-nuclear factor-κB feedback circuit modulates liver cancer progression. Hepatology.

[35] Darsigny M, Babeu JP, Seidman EG, Gendron FP, Levy E, Carrier J, Perreault N, Boudreau F (2010). Hepatocyte nuclear factor-4alpha promotes gut neoplasia in mice and protects against the production of reactive oxygen species. Cancer Res.

[36] Chang Hae Ryung, Nam Seungyoon, Kook Myeong-Cherl, Kim Kyung-Tae, Liu Xiuping, Yao Hui, Jung Hae Rim, Lemos Robert, Seo Hye Hyun, Park Hee Seo, Gim Youme, Hong Dongwan, Huh Iksoo (2014). HNF4α is a therapeutic target that links AMPK to WNT signalling in early-stage gastric cancer. Gut.

[37] McGee DJ, Mobley HL (2000). Pathogenesis of Helicobacter pylori infection. Curr Opin Gastroenterol.

[38] Tu S, Bhagat G, Cui G, Takaishi S, Kurt-Jones EA, Rickman B, Betz KS, Penz-Oesterreicher M, Bjorkdahl O, Fox JG, Wang TC (2008). Overexpression of interleukin-1beta induces gastric inflammation and cancer and mobilizes myeloid-derived suppressor cells in mice. Cancer Cell.

[39] Dinarello CA (1996). Biologic basis for interleukin-1 in disease. Blood.

[40] Shibata W, Takaishi S, Muthupalani S, Pritchard DM, Whary MT, Rogers AB, Fox JG, Betz KS, Kaestner KH, Karin M, Wang TC (2010). Conditional deletion of IkappaB-kinase-beta accelerates helicobacter-dependent gastric apoptosis, proliferation, and preneoplasia. Gastroenterology.

[41] Xiao H, Gulen MF, Qin J, Yao J, Bulek K, Kish D, Altuntas CZ, Wald D, Ma C, Zhou H, Tuohy VK, Fairchild RL, de la Motte C (2007). The Toll-interleukin-1 receptor member SIGIRR regulates colonic epithelial homeostasis, inflammation, and tumorigenesis. Immunity.

[42] Lamb Acacia, Chen Lin-Feng (2013). Role of the Helicobacter pylori-induced inflammatory response in the development of gastric cancer. J Cell Biochem.

[43] Hayden MS, Ghosh S (2008). Shared principles in NF-kappaB signaling. Cell.

[44] Kiselyuk A, Lee SH, Farber-Katz S, Zhang M, Athavankar S, Cohen T, Pinkerton AB, Ye M, Bushway P, Richardson AD, Hostetler HA, Rodriguez-Lee M, Huang L (2012). HNF4α antagonists discovered by a high-throughput screen for modulators of the human insulin promoter. Chem Biol.

[45] Fleischmann RM, Tesser J, Schiff MH, Schechtman J, Burmester GR, Bennett R (2006). Safety of extended treatment with anakinra in patients with rheumatoid arthritis. Annals of the rheumatic diseases.

