# Supplementary material for: Mutual amplification of HNF4α and IL-1R1 composes an inflammatory circuit in Helicobacter pylori associated gastric carcinogenesis
Source: Oncotarget. 2016 Feb 8;7(10):11349–63. doi: 10.18632/oncotarget.7239 (PMC4905478; doi:10.18632/oncotarget.7239)
Supplement: Supplementary file 1 [file oncotarget-07-11349-s001.pdf]

SUPPLEMENTARY TABLES AND FIGURES

Supplementary Table S1: PCR primers used in this study

**HNF4α**	Forward:5′-TGTCCCGACAGATCACCTC-3′
	Reverse:5′-CACTCAACGAGAACCAGCAG-3′
**P1 HNF4α**	Forward:5′-GAATGCGACTCTCCAAAACC-3′
	Reverse:5′-GGCACTGGTTCCTCTTGTCT-3′
**P2 HNF4α**	Forward:5′-GGGCTCCAGTGGAGAGTTC-3′
	Reverse:5′-CATAGCTTGACCTTCGAGTGC-3′
**IL-1R1**	Forward:5′-GATTCCTGCTATGATTTTCTC-3′
	Reverse:5′-AGTCTTCTGCTTTTCTTTACG-3′
**pGL3-P1HNF4α**	Forward:5′-GGTACCAATCATTTACCCAAGGTCCCA-3′
	Reverse:5′-AAGCTTCACCCCAAGTCAGGCATTCTA-3′
**pGL3-P2 HNF4α**	Forward:5′-GGTACCCCCTGGTTCAAACGATTCTC-3′
	Reverse:5′-AAGCTTCTCATAGTTTGCCCTGCTTC-3′
**pGL3-IL-1R1**	Forward:5′-GGTACCGCAAGGAGCCCAAGGTGT-3′
	Reverse:5′-AAGCTTCAGATCCCGAAGCAAACG-3′
**P1 HNF4α(ChIP)**	Forward:5′-GGGAACCGGGAAACTGCG-3′
	Reverse:5′-CCCACGATTTAGAAACCTA-3′
**P2 HNF4α(ChIP)**	Forward:5′-CCTCCCGTTTGCCACCA-3′
	Reverse:5′-CCTGTAACCCCAGCATT-3′
**Actin**	Forward:5′-AGTTGCGTTACACCCTTTCTTG-3′
	Reverse:5′-CACCTTCACCGTTCCAGTTTT-3′

Supplementary Table S2: Incidence of AG and expression of HNF4α in mice given MNU and infected with Hp

Group	Number of animals		Incidence of AG	High expression of HNF4α(No.)
	Initial	End		
**Control**	12	12	0	0
**MNU**	12	10	4	2
**MNU+Hp**	12	9	6	6

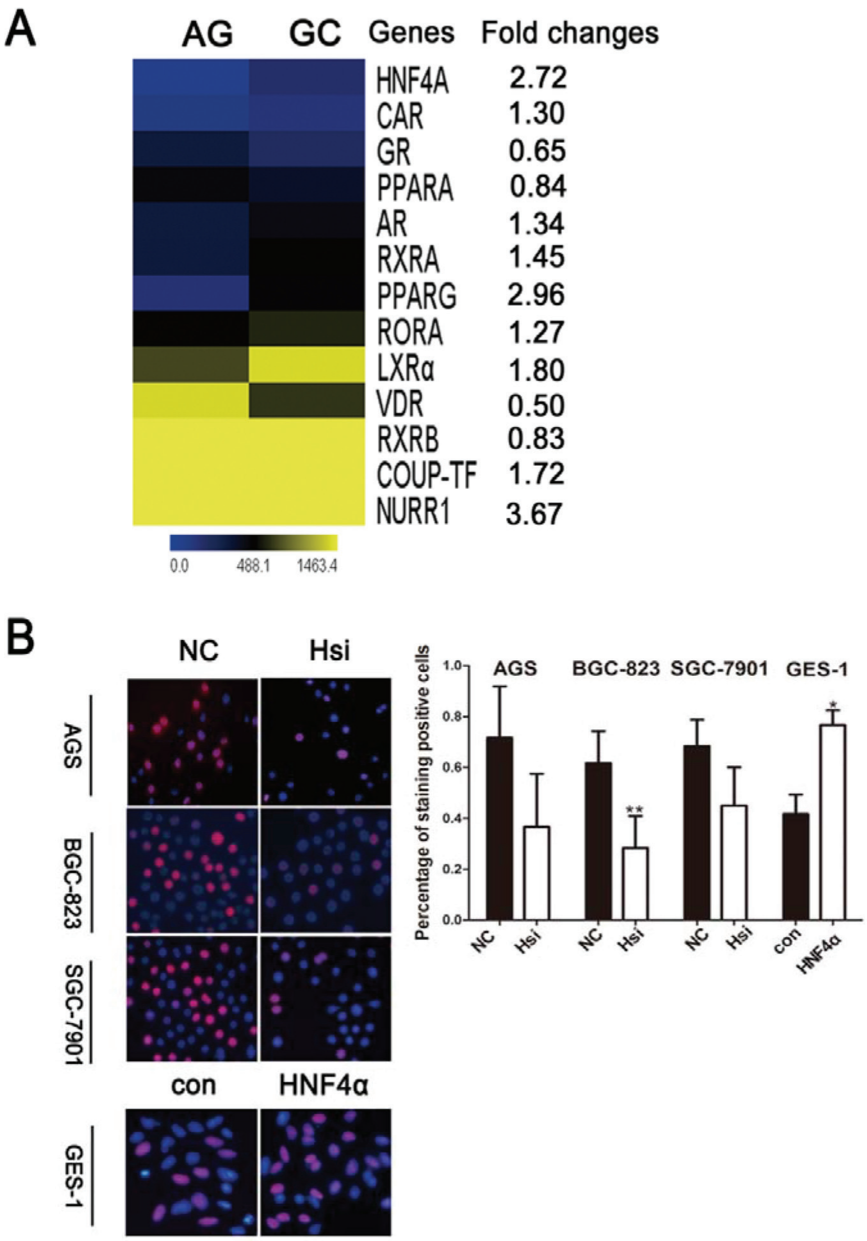

Supplementary Figure S1: A. Heat map shows expression changes of nuclear receptors in atrophic gastritis and gastric cancer. **B.** EdU staining after cells transfected HNF4α siRNA or HNF4α over-expression plasmid. Representative images are shown here from three independent biological replicates. *P< 0.05, **P< 0.01by Student's t test.

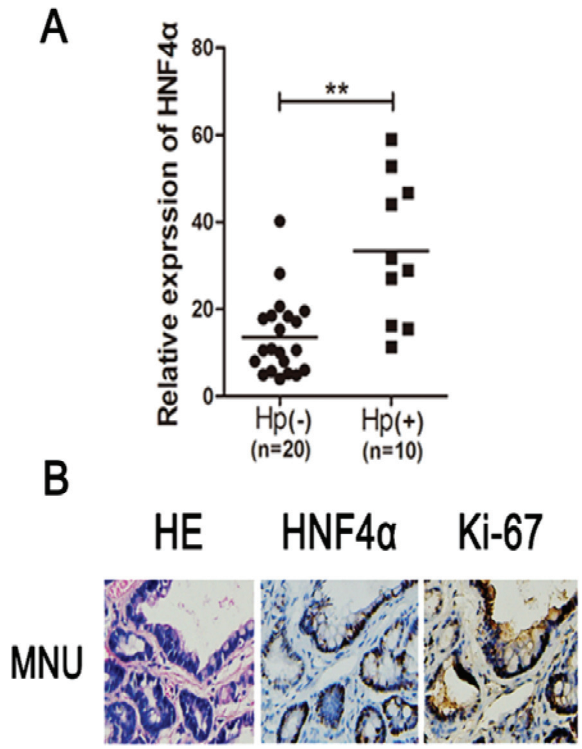

Supplementary Figure S2: A. The mRNA levels of HNF4α in 30 atrophic gastritis were measured by real-time PCR. The horizontal bars indicate the mean value of each sample group. **p<0.01 by Mann-Whitney U-test. **B.** HE staining and IHC of HNF4α and Ki-67 in MNU group.

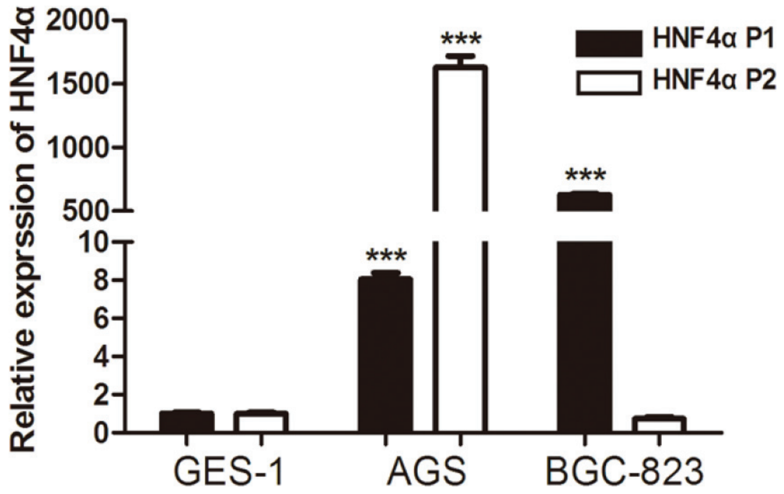

Supplementary Figure S3: Expression of P1- and P2-driven HNF4α in indicated cell lines using real-time PCR. ***P< 0.001by Student's t test.

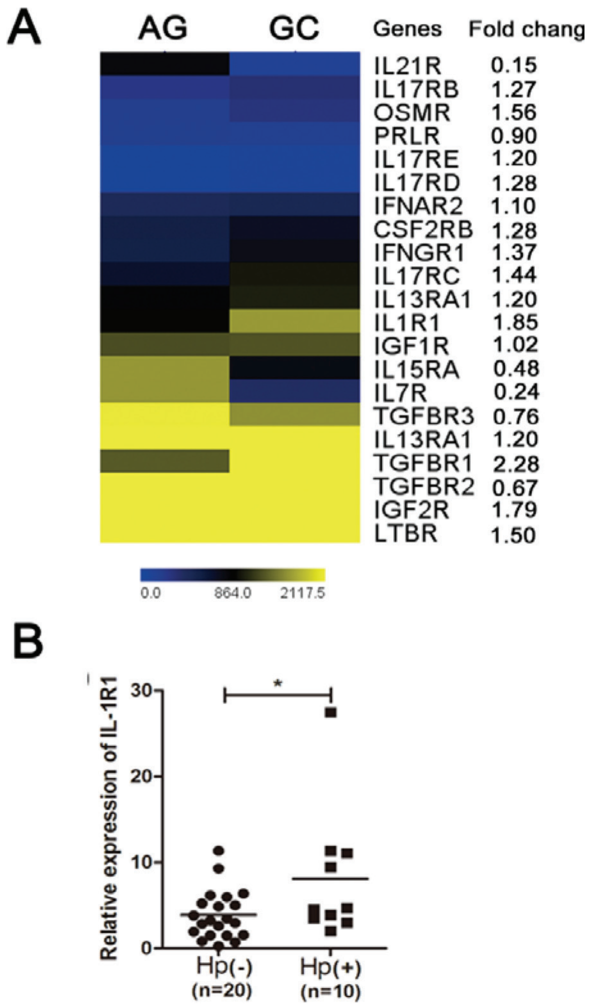

Supplementary Figure S4: A. Heat map shows expression changes of cellular surface receptors in atrophic gastritis and gastric cancer. **B.** The mRNA levels of IL-1R1 in 30 atrophic gastritis were measured by real-time PCR. The horizontal bars indicate the mean value of each sample group. *p<0.05 by Mann-Whitney U-test.

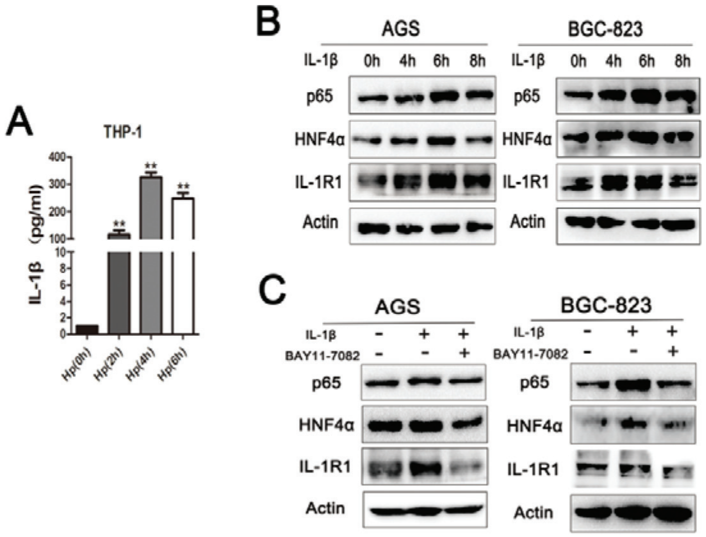

Supplementary Figure S5: A. Hp elicited IL-1βrelease by monocytic cell line THP-1. **P< 0.01by Student's t test. **B.** Western blot shows increased protein levels of p65, HNF4αand IL-1R1 when AGS and BGC-823 cells were treated with IL-1β (10ng/ml) for 4,6 and 8 hours. **C.** BAY 11-7082(1.0 mM) attenuated the induction of indicated protein expression by IL-1β.
